# Urinary Cyclophilin A as Marker of Tubular Cell Death and Kidney Injury

**DOI:** 10.3390/biomedicines9020217

**Published:** 2021-02-20

**Authors:** Ramio Cabello, Miguel Fontecha-Barriuso, Diego Martin-Sanchez, Ana M. Lopez-Diaz, Susana Carrasco, Ignacio Mahillo, Carmen Gonzalez-Enguita, Maria D. Sanchez-Niño, Alberto Ortiz, Ana B. Sanz

**Affiliations:** 1 Department of Urology, Hospital Universitario Fundación Jiménez Díaz, 28040 Madrid, Spain; rcabello@fjd.es (R.C.); cgenguita@fjd.es (C.G.-E.); 2 Research Institute-Fundación Jiménez Díaz, Autonoma University, 28040 Madrid, Spain; miguel.fontecha@quironsalud.es (M.F.-B.); diego.martin@fjd.es (D.M.-S.); anam.lopezd@quironsalud.es (A.M.L.-D.); scarrasco@fjd.es (S.C.); mdsanchez@fjd.es (M.D.S.-N.); 3 Department of Medicine, School of Medicine, Autonoma University, 28029 Madrid, Spain; 4 Department of Epidemiology and Biostatistics. Hospital Universitario Fundación Jiménez Díaz, 28040 Madrid, Spain; Imahillo@fjd.es; 5 Department of Pharmacology, Autonoma University, 28029 Madrid, Spain; 6 IRSIN (Instituto Reina Sofía de Investigacíon en Nefrología), 28003 Madrid, Spain

**Keywords:** acute kidney injury, tubular cell death, kidney transplantation, kidney cancer, ischemia-reperfusion

## Abstract

**Background:** Despite the term acute kidney injury (AKI), clinical biomarkers for AKI reflect function rather than injury and independent markers of injury are needed. Tubular cell death, including necroptotic cell death, is a key feature of AKI. Cyclophilin A (CypA) is an intracellular protein that has been reported to be released during necroptosis. We have now explored CypA as a potential marker for kidney injury in cultured tubular cells and in clinical settings of ischemia-reperfusion injury (IRI), characterized by limitations of current diagnostic criteria for AKI. **Methods:** CypA was analyzed in cultured human and murine proximal tubular epithelial cells exposed to chemical hypoxia, hypoxia/reoxygenation (H/R) or other cell death (apoptosis, necroptosis, ferroptosis) inducers. Urinary levels of CypA (uCypA) were analyzed in patients after nephron sparing surgery (NSS) in which the contralateral kidney is not disturbed and kidney grafts with initial function. **Results:** Intracellular CypA remained unchanged while supernatant CypA increased in parallel to cell death induction. uCypA levels were higher in NSS patients with renal artery clamping (that is, with NSS-IRI) than in no clamping (NSS-no IRI), and in kidney transplantation (KT) recipients (KT-IRI) even in the presence of preserved or improving kidney function, while this was not the case for urinary Neutrophil gelatinase-associated lipocalin (NGAL). Furthermore, higher uCypA levels in NSS patients were associated with longer surgery duration and the incidence of AKI increased from 10% when using serum creatinine (sCr) or urinary output criteria to 36% when using high uCypA levels in NNS clamping patients. **Conclusions:** CypA is released by kidney tubular cells during different forms of cell death, and uCypA increased during IRI-induced clinical kidney injury independently from kidney function parameters. Thus, uCypA is a potential biomarker of kidney injury, which is independent from decreased kidney function.

## 1. Introduction

Acute kidney injury (AKI) is a multifactorial and multiphasic kidney disease characterized by a rapid decline in kidney function, resulting in the accumulation of metabolic waste and toxins [1]. The main causes of parenchymal AKI include ischemia-reperfusion injury (IRI) and endogenous, as well as exogenous, nephrotoxins. Notwithstanding advances in preventive strategies and support measures, AKI continues to be associated with high morbidity and mortality. Despite its name, which includes the term “injury” rather than “failure”, current diagnostic criteria for AKI rely on evidence of decreased kidney function and not on evidence of kidney injury [1]. Decreased urine output or increased serum creatinine (sCr) may be observed in functional situations in the absence of kidney injury. As examples, sCr may increase, due to functional reasons, in the absence of injury when drugs that decrease the tubular secretion of creatinine are prescribed or when nephroprotective drugs decrease hyperfiltration, without concomitant kidney injury. Thus, biomarkers of actual kidney damage (injury) are needed for early diagnosis (before kidney function decreases), differential diagnosis (injury versus functional changes in the absence of injury) and the staging (e.g., is injury ongoing or not) of kidney injury that allows for early intervention and the adequate selection of therapeutic agents to improve outcomes.

Tubular cell death is a hallmark of parenchymal AKI [2,3,4,5]. Both apoptosis and regulated necrosis (RN) may contribute to tubular cell death and may be modulated therapeutically [4,5]. RN pathways include necroptosis, ferroptosis, mitochondria permeability transition-regulated necrosis, and pyroptosis, the later been a form of cell death in macrophages [3]. Tubular cell death is usually an early event during AKI that is followed by tubular regeneration. However, RN may trigger kidney inflammation that drives a second wave of tubular cell death, that contributes to maintaining decreased kidney function [6]. Indeed, we recently identified necroptosis as a key mode of cell death in experimental AKI and set out to explore potential biomarkers of necroptosis in the clinical setting. For that we identified a clinical scenario in which a highly controlled ischemia injury to the kidney occurred and in which traditional methods to assess kidney injury (based on functional changes) may not be informative, i.e., a clinical setting in which information provided by a marker of injury (rather than of function) may be clinically relevant.

Elective surgical aggression to the kidney, such as nephron sparing surgery (NSS) with renal artery clamping and tumor resection, or kidney transplantation (KT) with anoxia, hypothermia and reperfusion represent clinical practice models of kidney IRI that may or may not result in clinical AKI as per the current consensus definition based on functional assessment [7]. Thus, in NSS, the contralateral kidney is not disturbed and may provide sufficient urine volume or glomerular filtration rate (GFR) to limit or prevent a decrease in diuresis or increase in sCr, despite histological tubular necrosis related to ischemia, and thus, patients may not fulfill current (functional) criteria to diagnose AKI. In KT, despite ischemic injury to the graft, the injured graft may still provide better GFR that the non-functioning native kidneys, and diuresis and decreasing sCr may be observed in the presence of histological kidney injury, unless the graft injury is severe, leading to primary non-function. The ability to assess AKI by noninvasive tools before kidney function declines, with the accurate categorization of severity and the possibility to identify different causes may improve the functional outcome after kidney surgery.

Cyclophilin A (CypA) is the most abundant and ubiquitous member of the cyclophilin family of four proteins (A, B, C, and D) [8,9]. CypA is an 18 kDa intracellular protein with roles in protein folding, trafficking, and T-cell activation [8,10,11,12]. CypA has been reported to be actively released to the extracellular milieu by injured cells and extracellular CypA could activate inflammation and proliferation [13]. Specifically, cytosolic CypA is released early in necroptosis, and has been proposed to be a biomarker for this type of cell death when the integrity of the plasma membrane is compromised [14]. High levels of CypA are found in the kidney [15,16]. Stressed proximal tubular cells release CypA; however, the relationship of this release to tubular cell death was not previously studied. In this regard, urinary CypA (uCypA) was reported to be increased in diabetic kidney disease, a condition associated with tubular cell death, increased glomerular permeability and proximal tubular dysfunction [17,18]. CypA can be freely filtered by kidney glomeruli; therefore, higher levels in urine samples may reflect an increase in serum levels or elevated excretion by injured kidney tubular cells. In this regard, both serum and urine CypA were higher in patients with postoperative AKI after cardiac surgery and, thus, increased serum CypA appears to be a biomarker of deceased kidney function [19].

Thus, we set out to explore whether uCypA could be a marker of tubular cell necroptosis in the clinic. Now, we report that CypA is released by proximal tubular kidney cells exposed to different lethal stimuli that induce diverse forms of cell death. Moreover, uCypA increased in clinical situations of kidney injury even when there was no associated functional impairment and it better associated with clinical evidence of more severe kidney insult than the more classical biomarker NGAL. We, therefore, identify uCypA as a potential biomarker of kidney injury independent from reduced kidney function.

## 2. Materials and Methods

### 2.1. Cell Culture 

Two tubular cell lines were studied, human (HK-2) [20] and murine (MCT) [21] immortalized proximal tubular epithelial cell lines. HK-2 cells were grown in RPMI 1640 (Thermo Fisher, Waltham, MA, USA), 10% decomplemented fetal bovine serum (FBS) (Sigma-Aldrich-Merck KGaA, Damstadt, Germany), 1% glutamine, 100 U/mL penicillin, 100 µg/mL streptomycin, 5 μg/mL Insulin Transferrin Selenium (ITS) and 36 ng/mL hydrocortisone in 5% CO_2_ at 37 °C. MCTs were grown in RPMI 1640, 10% FBS, 100 μg/mL streptomycin, 2 mM glutamine, and 100 U/mL penicillin. At 60–70% of confluence, cells were growth-arrested in serum-free medium for 24 h before the experiments. 

HK2 were exposed to the chemical hypoxia inducer CoCl_2_ (Merck, Darmstadt, Germany) at 500 µM and to the ferroptosis inducer RSL3 (SelleckChem, Houston, TX, USA) at 400 nM. MCT cells were exposed to lethal cytokine cocktail that induces apoptosis (TTI), consisting of 100 ng/mL TWEAK (Merck), 30 ng/mL TNFα (PeproTech, Rocky Hill, NJ, USA) and 30 U/mL interferonγ (PeproTech, Rocky Hill, NJ, USA), to which 25 µM pan-caspase inhibitor carbobenzoxy-valyl-alanyl-aspartyl-[O-methyl]-fluoromethylketone (TTI+zVAD) (Bachem, Bubendorf, Switzerland) may be added to switch the mode of cell death to necroptosis [6,22]. To perform hypoxia/reoxygenation (H/R) experiments, MCT cells were cultured for 24 h in a hypoxic atmosphere containing 1% O_2_, 94% N_2_, 5% CO_2_ (Air Liquide, Madrid, Spain) in serum-free RPMI medium, after cells were maintained in 21% O_2_ for reoxygenation for indicated periods of time [23]. 

### 2.2. Western Blot

After the removal of cell culture media, cells were trypsinized, collected and homogenized in lysis buffer (50 mM TrisHCl, 150 mM NaCl, 2 mM EDTA, 2 mM EGTA, 0.2% Triton X-100, 0.3% NP-40, 1 mM PMSF and 1 μg/mL pepstatin A) [24]. Protein concentration was measured with the BCA (bicinchoninic acid) assay (Thermo Fisher, Waltham, MA, USA). Cell supernatants were centrifuged at 2500 rpm, for 20 min at 4 °C to remove cells and debris, and then stored at −80 °C until analysis. Equal amounts of protein were loaded in 15% SDS gels, separated by electrophoresis and transferred to PVDF membranes (polyvinylidene difluoride, Millipore, Darmstadt, Germany). The membranes were blocked with 5% TBS/0.5% *v*/*v* Tween-20 skim milk and incubated with anti-cyclophilin A (1:500, Abcam) and anti-HIF-1α (1:2000, Abcam) antibodies dissolved in 5% milk TBS/Tween for 1 h at room temperature. They were then washed with TBS/Tween and incubated with the secondary antibodies against rabbit IgG (1:5000) or mouse IgG (1:5000) from GE Healthcare. After washing with TBS/Tween, blots were developed with the chemiluminescence method (ECL; Fisher Scientific, Waltham, MA, USA) and probed with mouse monoclonal anti-α-tubulin antibody (1:10,000; Sigma-Aldrich KGaA, Damstadt, Germany) for HIF membranes or anti-GAPDH (1:5000, Sigma-Aldrich) for CypA membranes, as these markers were allowed to run the gels to optimally separate the bands, given the different molecular weights of the proteins. Levels of expression were corrected for minor differences in loading. Cell supernatants were corrected with red ponceau staining. 

### 2.3. Cell Viability Assay

Cell viability was estimated using the 3-[4,5-dimethylthiazol-2-yI]-2,5 diphenyltetrazolium bromide (MTT, Sigma Aldrich-Merck KGaA, Damstadt, Germany) colorimetric assay. Following stimulation, culture medium was removed, and cells were incubated with 0.5 mg/mL MTT in PBS for 1 h at 37 °C. The resulting formazan crystals were dried and dissolved in DMSO. Absorbance (indicative of cell viability) was measured at 570 nm. 

### 2.4. NSS and KT Patients 

This observational study prospectively collected urinary samples and clinical data at the Fundación Jiménez Díaz University Hospital biobank between March 2014 and June 2019. The IIS-FJD institutional review board approved the study and patients signed and informed consent, approval date: 20 February 2019 A total of 64 patients were studied: 52 patients with a localized kidney tumor to whom a laparoscopic NSS was proposed and 6 donor-recipient pairs of living KT patients (laparoscopic living donor nephrectomy and conventional KT). Patients were retrospectively divided into 3 groups: laparoscopic NSS patients without (*n* = 13) or with (*n* = 39) arterial clamping during the procedure, and transplant (*n* = 6) patients with end-stage kidney disease who received a KT after laparoscopic nephrectomy of a living related donor. 

NSS patients had a healthy contralateral kidney. Medical background, tumor characteristics, surgical findings, warm ischemia time (WIT) when applied and postoperative evolution were recorded. Minimally invasive NSS involves laparoscopic approach and arterial control with a tourniquet. The final decision on clamping the renal artery was made by the surgeon according to the surgical situation (tumor limit visualization, bleeding or expected WIT). Diuretics, such as furosemide and mannitol, were not used.

Kidney donor candidates were studied according to local protocols and donated the left kidney. Kidneys were retrieved for transplantation by a laparoscopic hand-assisted approach. WIT and cold ischemia time (CIT) were recorded. 

Basal analytical data were obtained from preoperative blood testing. Postoperative lab tests were obtained from routine clinical lab testing during hospitalization. The last blood test available was also recorded during follow up. Urine samples were obtained serially: before surgery, upon bladder catherization at the time of anesthesia induction (baseline determination, Pre-Qx), immediately after surgery, before patients were woken up from anesthesia (0 h determination), the day after surgery (24 h) and daily thereafter (48 h, 72 h) up to 72 h while they were hospitalized. 

### 2.5. Urine Samples Collection and ELISA

Urine samples were collected in sterile containers and centrifuged at 2500 rpm for 20 min at 4 °C to remove cells and debris. Supernatants were stored at −80 °C until analysis. uCypA and urinary NGAL (uNGAL) were measured by an enzyme linked immunosorbent assay (ELISA, MyBiosource, San Diego, CA, USA and Bioporto, Hellerup, Denmark, respectively), according to the manufacturer specification. uCypA and uNGAL were adjusted to urine creatinine levels and expressed per mg of urinary creatinine, as is routinely done for other urinary analysis, such as albuminuria and proteinuria to account for potential physiological differences in urine dilution.

### 2.6. Statistics

Results are expressed as mean ± SD in cultured cells and as mean ± SEM or median (interquartile range) for patient data. Differences between groups were evaluated using one-way ANOVA with Tukey’s post hoc tests using the Prism software (Graphpad 7.04). For pairs of samples, data were analyzed using Student’s t test. Differences between non-clamping and clamping NSS patients were assessed by Student´s t test for normally distributed variables or by Wilcoxon rank test for non-normally distributed variables. Differences between categorical values were assessed by Fisher’s exact test. A *p*-value < 0.05 was considered statistically significant.

## 3. Results

### 3.1. Lethal Stimuli Promote CypA Release from Cultured Kidney Tubular Cells

Tubular cells represent that largest cell mass in the kidney and are key targets in parenchymal AKI during IRI. We focused on proximal tubular cells for cell culture studies to assess CypA release to cell culture media following exposure to lethal stimuli. Lethal stimuli were tested in cultured human or murine cells according to their sensitivity to specific modes of death.

**Figure 1:**
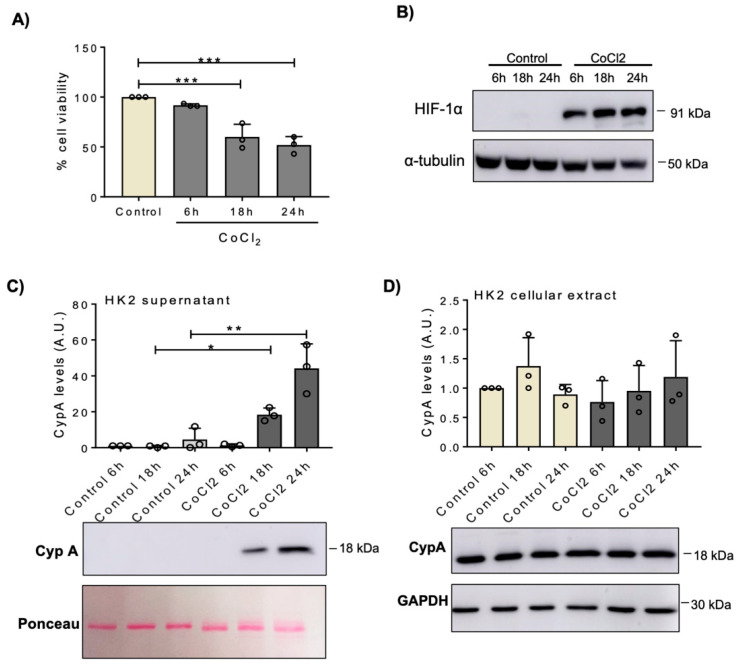
CypA is released by cultured human proximal tubular HK2 cells under chemical hypoxia. HK2 cells were exposed to 500 µM CoCl_2_ for the indicated periods of time. (**A**) Cell viability was assessed by the MTT assay. (**B**) HIF-1α expression, a marker of hypoxia, was assessed by Western blot. (**C**,**D**) Western blot of CypA in cell supernatants (**C**) and in cellular extracts (**D**). Quantification and representative image. (**A**,**C**,**D**) Mean ± SD of three independent experiments. * *p* < 0.05; ** *p* < 0.01; *** *p* < 0.001. Uncropped gel scans shown in Figure S1A.

**Figure 2:**
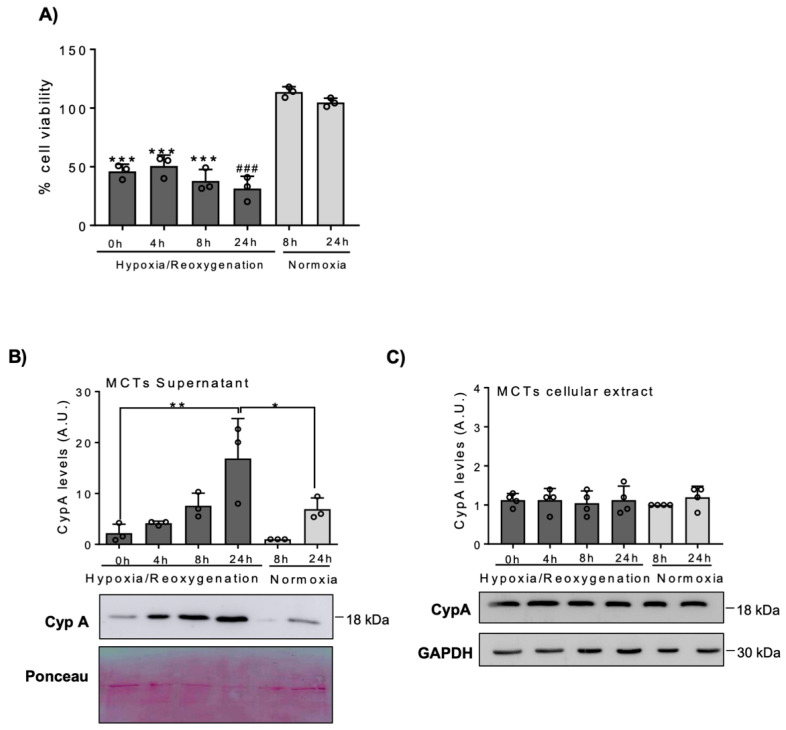
CypA is released by cultured murine proximal tubular MCT cells under hypoxia/reoxygenation. MCT cells were cultured for 24 h in a hypoxic atmosphere, and then cells were reoxygenated for the indicated periods of time (**A**) Cell viability was assessed by the MTT assay. Mean ± SD of three independent experiments. *** *p* < 0.001 vs. normoxia 8 h; ### *p* < 0.001 vs. normoxia 24 h. (**B**,**C**) Western blot of CypA in cell supernatants (**B**) and in cellular extracts (**C**). Quantification and representative image. Mean ± SD of three independent experiments. * *p* < 0.05; ** *p* < 0.01. Uncropped gel scans shown in Figure S1B.

Hypoxic conditions were simulated by the exposure of human proximal tubular cells (HK-2) to 500 µM CoCl_2_ for different periods of time. CoCl_2_ decreased cell viability (Figure 1A) and increased the hypoxia marker HIF-1α, as assessed by Western blot (Figure 1B) in a time-dependent manner. Under these conditions, CypA released into the cell culture media increased in a time-dependent manner, in parallel to the decreased cell viability (Figure 1C), while intracellular levels of CypA in adherent cells remained unchanged (Figure 1D). Murine proximal tubular cells (MCT cells) were exposed to H/R resulting in a time-dependent decreased cell viability, as confirmed by MTT assay (Figure 2A), which was paralleled by a progressive increase in extracellular CypA (Figure 2B), again without changes in intracellular CypA (Figure 2C).

**Figure 3:**
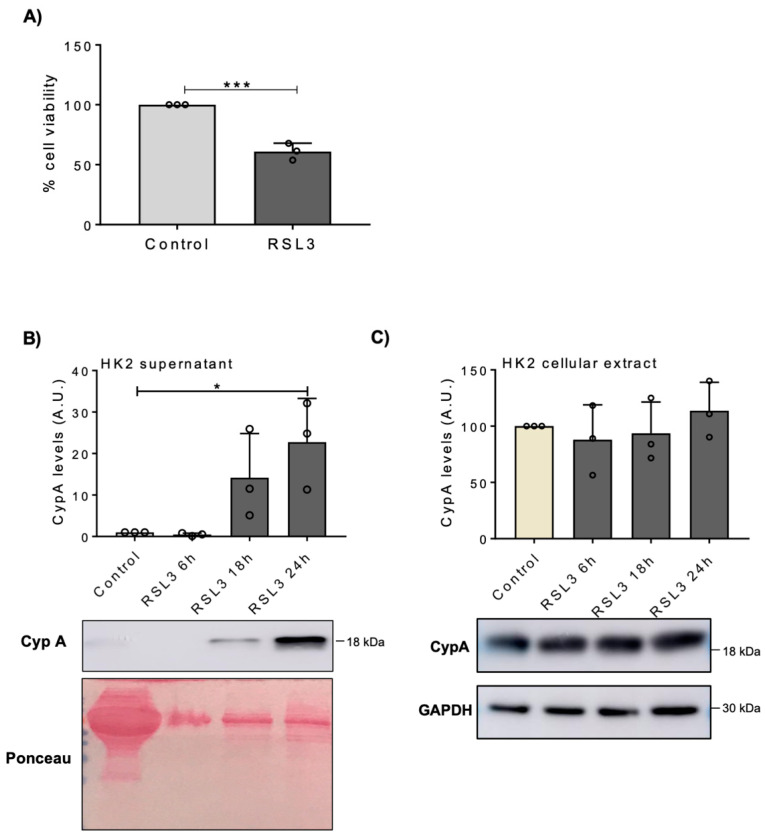
Ferroptosis leads to CypA release in human proximal tubular HK2 cells. (**A**) HK2 cells were exposed to 400 nM RSL3, a ferroptosis inducer, for 24 h and cell viability was assessed by the MTT assay. Mean ± SD of three independent experiments. *** *p* < 0.001. (**B**,**C**) HK2 cells were exposed to 400 nM RSL3 for indicated periods of times and CypA was measured by Western blot in cellular supernatants (**B**) and in cellular extracts (**C**)**.** Quantification and representative image. Mean ± SD of three independent experiments. * *p* < 0.05. Uncropped gel scans shown in Figure S2A.

**Figure 4:**
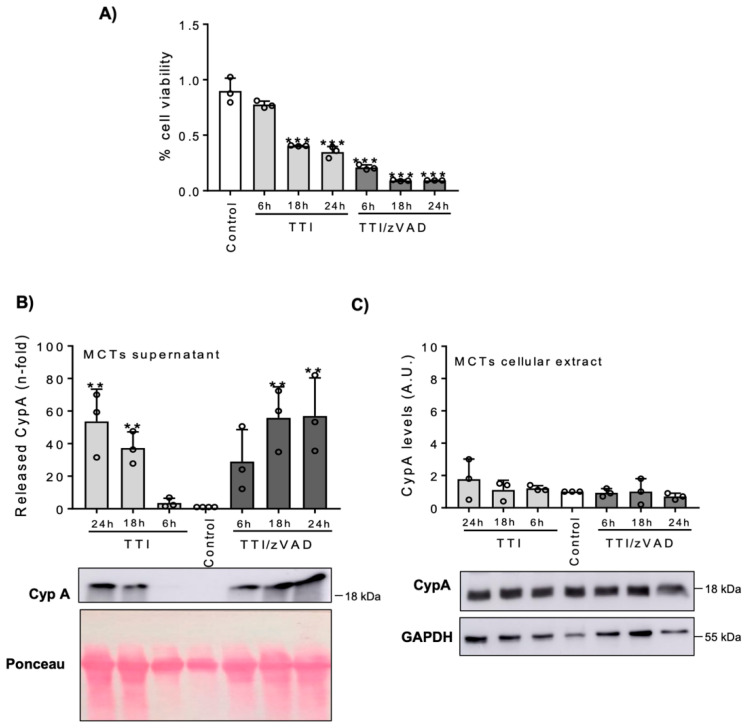
MCT cells were exposed to the apoptosis inducer cytokine cocktail TWEAK/TNFα/interferon-γ (TTI) or to the necroptosis inducer TTI/zVAD for the indicated periods of time. (**A**) Cell viability was assessed by the MTT assay. Mean ± SD of three independent experiments. *** *p* < 0.001 vs. control. (**B**,**C**) Western blot of CypA in cell supernatants (**B**) and in cellular extracts (**C**). Note than controls are in the middle of the gel and the time course is represented from there, i.e., later time points for both stimuli are at opposite sites of the gel and figure. Quantification and representative image. Mean ± SD of three independent experiments. ** *p* < 0.01 vs. control. Uncropped gel scans shown in Figure S2B.

To address whether CypA release is linked to any specific pathways of cell death, tubular cells were exposed to specific inducers of different cell death pathways. Ferroptosis was induced in HK-2 by exposure to RSL3, which resulted in decreased cell viability by 24 h, as tested by the MTT assay (Figure 3A). RSL3 also increased CypA release to the cell culture media (Figure 3B), without changes in intracellular CypA (Figure 3C). Apoptosis and necroptosis were induced in MCT cells by exposure to TTI and TTI/zVAD, respectively. Both stimuli reduced cell viability (Figure 4A), and increased CypA release (Figure 4B), without changes in intracellular CypA (Figure 4C). However, the temporal pattern of CypA release differed between apoptosis and necroptosis, as expected from the specific characteristics of each form of cell death [22]. Thus, as other forms of RN, necroptosis is characterized by loss of cell membrane integrity and release of intracellular contents, and this was consistent with an earlier release of CypA (Figure 4B). By contrast, apoptotic cells do not initially release cellular contents, but in culture they are not engulfed by macrophages or adjacent uninjured cells, and eventually the plasma membrane becomes permeabilized.

Overall, these results suggest that CypA is released by kidney tubular cells exposed to lethal stimuli and CypA release is independent of the mechanism of cell death.

### 3.2. Clinical Study: Patient Characteristics

The clinical study was performed in two different clinical settings, both characterized by limitations in the current KDIGO definition of AKI to identity kidney injury: 52 patients undergoing NSS (13 with no-clamping and 39 with clamping of renal artery) and 6 living donor-kidney transplant recipient pairs.

Among NSS patients (Table 1), no significant differences were found between patients with or without renal artery clamping regarding age, gender, body mass index, cardiovascular risk factors, cardiovascular events, kidney function or the nephrometric scores RENAL, PADUA, C. Index, ABC, RPS, or Mayo (Supplementary Table S1). However, tumor size (22.5 ± 8.93 mm vs. 31.3 ± 8.93 mm, *p* = 0.006), DAP (5 (2.00) vs. 6 (2.00), *p* = 0.021) and NePhRO (6 (2.00) vs. 8 (2.00), *p* = 0.011) scores were higher in the NSS patients with renal artery clamping. The WIT in NSS patients with arterial clamping was 23.83 ± 8.38 min.

Table 2 summarizes the characteristics of 6 pairs of KT donors and recipients. Donor laparoscopic hand-assisted left nephrectomy was followed by uneventful graft transplantation in the right iliac fossa, immediate diuresis and no need for postoperative dialysis.

### 3.3. Renal Artery Clamping during NSS Is Associated with Increased uCypA

**Figure 5:**
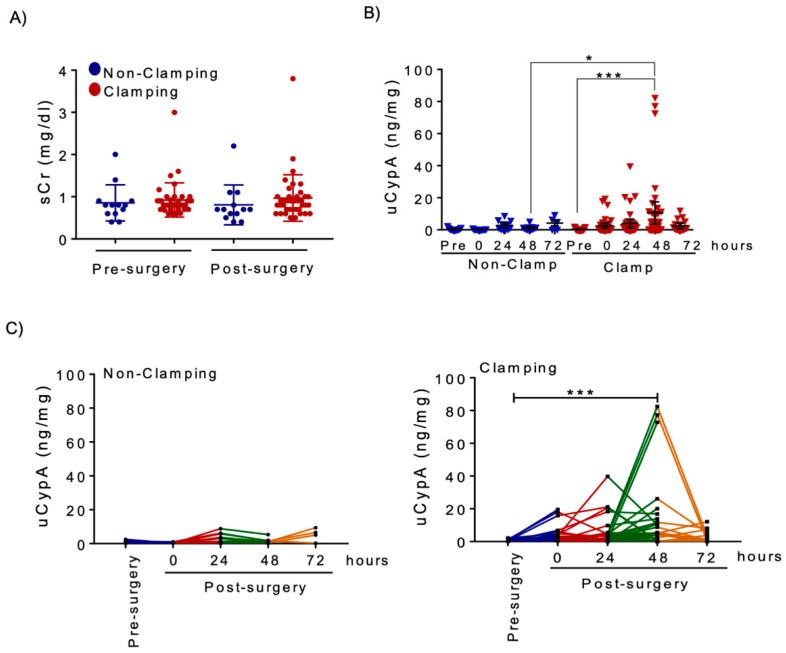
uCypA levels are increased in NSS patients with renal artery clamping but not in patients without clamping. (**A**) sCr levels after surgery did not differ between clamping and non-clamping patients. (**B**) uCypA levels are increased after surgery in NSS patients with clamping. Bars represent mean ± SEM. * *p* < 0.05; *** *p* < 0.001. (**C**) Time course of uCypA levels in individual patients shows a peak at 24–48 h after surgery in most clamping patients, while no clear peak was observed in non-clamping patients. (**B**,**C**) uCypA is expressed as urinary CypA: urinary creatinine ratio.

Among NSS patients, five met the increased sCr KDIGO diagnostic criterion for AKI stage I after surgery, 4 (10.25%) in the clamping group and 1 (7.69%) in the non-clamping group (p ns) (Table 1, Figure 5A).

Next, we measured uCypA levels and stratified NSS patients according to renal artery clamping, which represents a kidney IRI. uCypA levels did not significantly change over time in non-clamping patients and did not increase above baseline in the single non-clamping patient that fulfilled AKI KDIGO criteria. By contrast, uCypA was already increased immediately after surgery (uCypA values were between 14-fold and 33-fold higher than baseline in three patients at this point) in some clamping patients and mean values further increased up to 48 h after surgery, at which point they were significantly higher than at baseline (10.58 ± 3.38 vs. 0.39 ± 0.08 ng/mg, *p* < 0.001) (Figure 5B). Individual clamping patients usually showed peak uCypA levels at 24–48 h, while no clear peak was observed in non-clamping patients (Figure 5C). uCypA increased from baseline in the four clamping patients who fulfilled the KDIGO AKI sCr criterion and, additionally in 15 further patients for whom increased uCypA provided evidence of kidney injury in the absence of clinically relevant decrease in kidney function, as assessed by sCr levels. The low sensitivity of KDIGO AKI criteria to detect AKI in this clinical setting was expected, given that the contralateral kidney was not disturbed by surgery.

**Figure 6:**
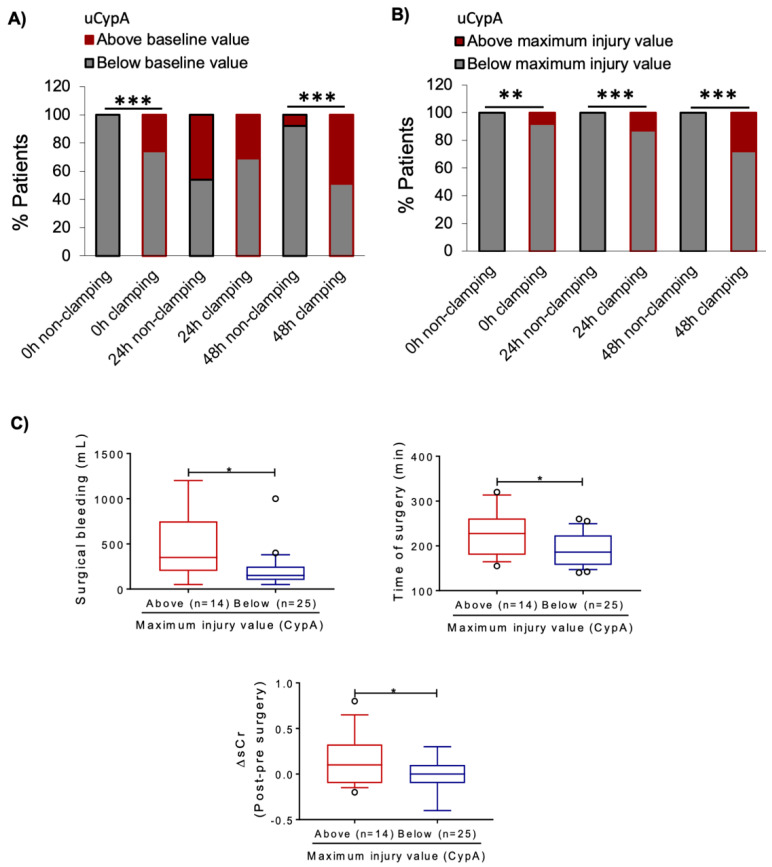
Percentage of patients with high uCypA levels after NSS using two different uCypA cut-off levels to define “high uCypA”. (**A**) Percentage of patients with post-surgery uCypA values above the highest pre-surgery uCypA value, found in the study population, i.e., >25 ng/mg; *** *p* < 0.001. (**B**) Percentage of patients with post-surgery uCypA values above the highest post-surgery uCypA values obtained in NSS without clamping, i.e., >100 ng/mg; ** *p* < 0.01, *** *p* < 0.001. (**C**) Time of surgery, surgical bleeding and increase in sCr are higher in clamping patients with CypA above maximum injury levels. Data are presented as box plots that show the mean, 25/75 percentiles (box), and 10/90 percentiles (bars). * *p* < 0.05.

To gain insight into potential cut-off points of uCypA that may be used to define the occurrence of kidney injury, post-surgery uCypA values were compared to two different cut-off points. Clamping resulted in a higher percentage of patients above both cut-off points (Figure 6A,B). First, the cut-off point for uCypA was defined as post-surgery values above the highest pre-surgery uCypA value found in the study population. This uCypA cut-off point was labeled as an increase above baseline value (>2.5 ng/mg, Figure 6A). Using this definition, an immediate increase in uCypA values was observed in 26% of clamping patients but in none of the no-clamping patients (Figure 6A). However, by 24 h, increased uCypA was observed in 31% and 46% of clamping and no-clamping patients and this difference was not statistically significant. By 48 h, uCypA values above the baseline value cut-off point were observed almost exclusively in clamping patients (49%). However, 24-h findings suggested that surgery-associated tissue injury, unrelated to kidney IRI, could result in mild increases in uCypA in the early post-surgery period. Thus, a second uCypA cut-off point was defined as post-surgery values above the highest uCypA values obtained within 72 h of surgery without clamping, this was named the maximum injury value cut-off point (i.e., >10 ng/mg, Figure 6B) and accounted for non-specific surgery-induced tissue injury, unrelated to IRI. Using this definition, only clamping resulted in uCypA values above the cut-off point and the percentage of patients with significant kidney injury potentially linked to IRI increased from 8% immediately post-surgery to 28% at 48 h (Figure 6B). A total of 14 (36%) patients had a higher uCypA at some point after surgery. Moreover, patients with uCypA above the maximum injury cut-off point showed longer surgery duration, and higher surgical bleeding and increase in sCr after surgery (Figure 6C), suggesting that high levels of CypA are related to more severe kidney injury. In this regard, the four clamping patients with clinical AKI had uCypA above baseline value cut-off point and three of them went above the maximum injury cut-off point.

Overall, these findings suggest the potential usefulness of uCypA to detect IRI kidney injury when function parameters are unreliable.

### 3.4. Urine NGAL in NSS Patients

Next, we assessed a conventional kidney injury biomarker, uNGAL, in NSS patients. Among the five NSS patients meeting the increased sCr KDIGO diagnostic criterion for AKI stage I after surgery, only one (20%), belonging to the clamping group, had an increase in uNGAL above baseline.

**Figure 7:**
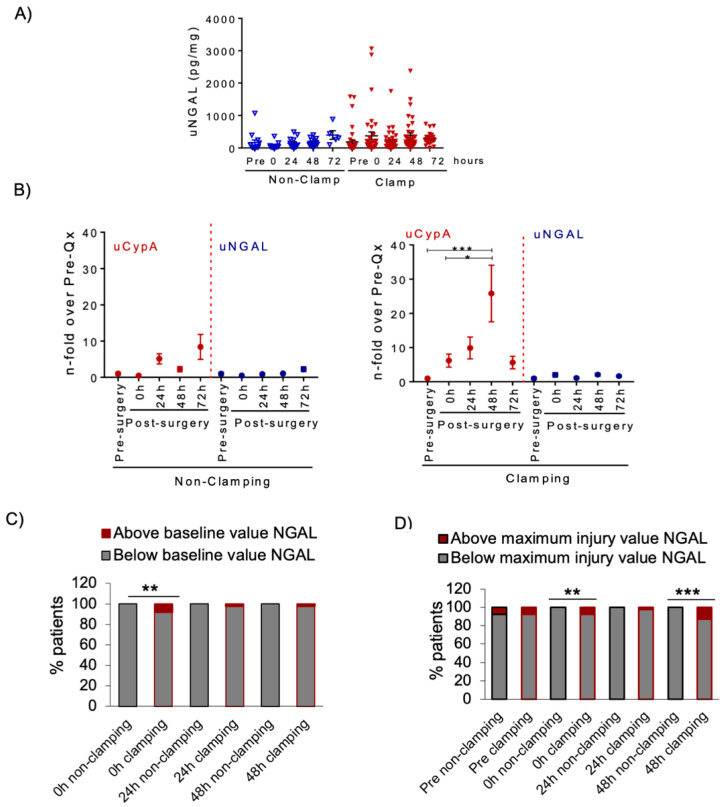
uNGAL levels in NSS patients. (**A**) uNGAL levels were measured in NSS patients before and after surgery and no statistically significant changes were observed. uNGAL is expressed as urinary NGAL: urinary creatinine ratio. Bars represent mean ± SEM. (**B**) Time-course of uNGAL and uCypA values expressed as fold-change over mean baseline pre-surgery values. Statistically significant increases over baseline were only observed for uCypA in clamping patients. Mean ± SEM, * *p* < 0.05; *** *p* < 0.001. (**C**) Percentage of patients with post-surgery uNGAL values above the highest pre-surgery uNGAL value found in the study population, i.e., >16 pg/mg. (**D**) Percentage of patients with post-surgery uNGAL values above the highest post-surgery uNGAL values obtained in NSS without clamping, ** *p* < 0.01, *** *p* < 0.001.

As for uCypA, clamping resulted in numerically higher mean uNGAL values at 48 h when compared to baseline (392 ± 81 vs. 187 ± 66 pg/mg) than in non-clamping compared to baseline (167 ± 82 vs. 174 ± 32 pg/mg); however, these differences were not statistically significant (Figure 7A). Indeed, the fold-change over the mean baseline values was lower for uNGAL than for uCypA and no statistically significant differences were observed in uNGAL in either clamping or no-clamping patients (Figure 7B). This differs from the significant (48 h *p* < 0.001) and sustained mean 6.4- to 26.5-fold increase in uCypA versus pre-surgery values in clamping patients (Figure 7B).

Use of the same definitions (increase above baseline cut-off and maximum injury cut-off values) for high uNGAL levels as previously used for uCypA did not disclose a clear association of high uNGAL levels with either surgery or clamping for any of the definitions (Figure 7C). While using the above maximum injury cut-off (i.e., above peak post-surgery value in non-clamping cut-off value) resulted in a significantly higher percentage of clamping patients with high uNGAL levels, this percentage (12.5%) was in the range observed at baseline for both clamping and non-clamping patients (Figure 7D). Finally, contrary to uCypA, there was no clear association between uNGAL values above the maximum injury cut-off point with surgery duration or increased sCr after surgery (Figure S3).

The correlation between uCypA and uNGAL was poor and only statistically significant for clamping patients (Figure S4). Overall, these results suggest that uNGAL appears less sensitive than uCypA to detect IRI-induced kidney injury.

### 3.5. Urine CypA Levels Following Living Donor KT

**Figure 8:**
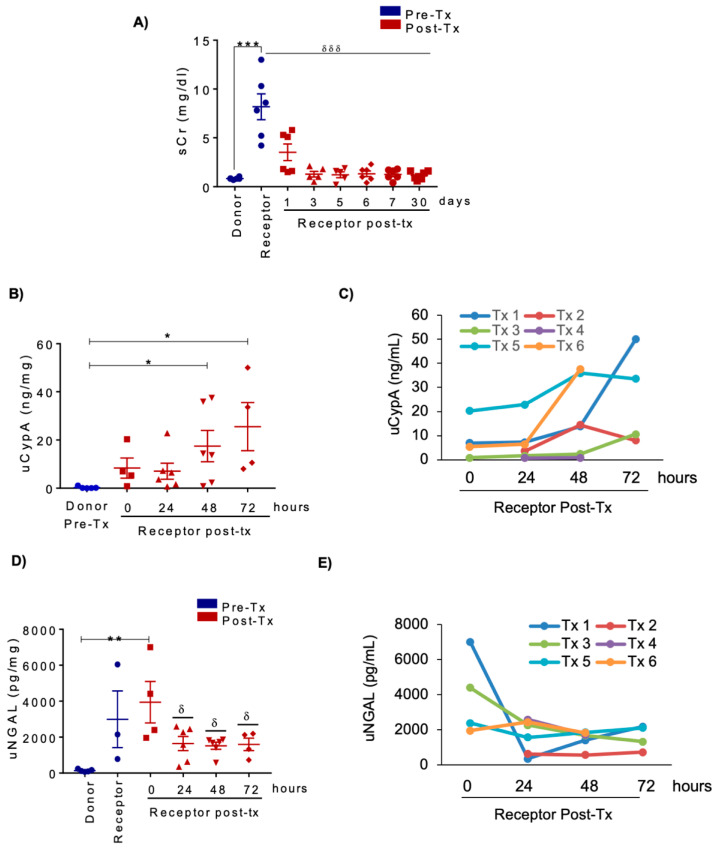
uCypA levels are increased after living KT. (**A**) sCr levels decreased following KT. Bars represent mean ± SEM. *** *p* < 0.05 vs. donor; δδδ *p* < 0.001 vs. receptor pre-transplant. (**B**) By contrast, uCypA levels increased following KT in recipients compared with donor urine. Bars represent mean ± SEM. * *p* < 0.05. uCypA is expressed as urinary CypA: urinary creatinine ratio. (**C**) Time-course of uCypA levels in individual patients shows a peak at 24–48 h after surgery in most patients. (**D**) uNGAL levels decreased from time zero after surgery in KT recipients. uNGAL is expressed as urinary NGAL: urinary creatinine ratio. Bars represent mean ± SEM. ** *p* < 0.01. (**E**) Time-course of uNGAL levels in individual patients shows a decrease after surgery in most patients.

We next analyzed uCypA in KT patients after living kidney donation with immediate diuresis and kidney function and progressive normalization of sCr in recipients (Figure 8A). Mean uCypA levels in transplanted patients increased progressively from time zero as compared with donor urine, reaching statistical significance at 48 and 72 h (Figure 8B,C). In contrast, peak uNGAL was observed at time zero, the only time point in which it was significantly higher than in donor urine, and uNGAL decreased thereafter (Figure 8D,E). In this regard, pre-surgery uNGAL values in KT recipients were above those in donors and in the range found in recipient urine at time zero, suggesting that part of this uNGAL originated from the recipient kidneys. This is not surprising, since CKD has been associated with increased uNGAL [33].

Overall, these results further support the potential usefulness of uCypA to detect IRI kidney injury when function parameters are unreliable.

## 4. Discussion

**Figure 9:**
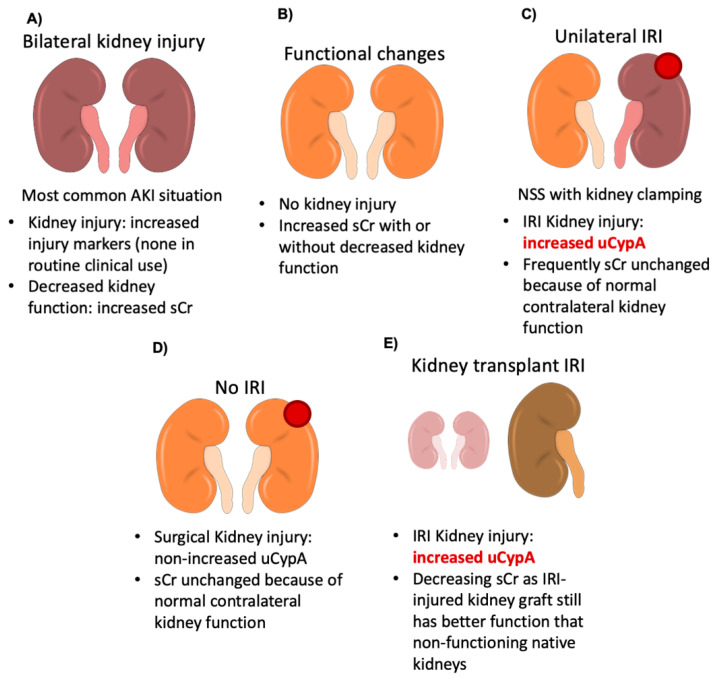
Conceptual figure: current issues in AKI diagnosis and contribution of uCypA. (**A**) Current AKI diagnosis. (**B**) sCr may increase due to functional reasons, in the absence of injury. (**C**) Unilateral IRI resulting from clamping of renal artery in NSS. Increased uCypA in IRI kidney injury. uCypA was a more sensitive marker of IRI-induced kidney injury than sCr or classical markers of injury such as NGAL. (**D**) In NSS without clamping, there is no IRI. (**E**) In KT patients, kidney graft IRI is usually associated with decreasing sCr levels. Increased uCypA in IRI kidney injury. Again, uCypA provided evidence of kidney injury despite decreasing sCr and non-informative uNGAL.

The main findings are that CypA is released by dying tubular cells and the release is not specific for any form of cell death (apoptosis, necroptosis, ferroptosis), thus, potentially identifying the ongoing kidney injury of diverse etiology. In this regard, using clinical situations in which sCr or diuresis do not adequately represent underlying IRI-induced kidney injury, we have identified uCypA as a marker of clinical IRI kidney injury more sensitive that the classical markers uNGAL or sCr to detect unilateral kidney injury in presence of a functional contralateral kidney or when kidney injury nonetheless results in better kidney function than in the baseline situation, as is the case for KT (Figure 9). While these clinical situations allowed us to identify the role of uCypA as a marker of kidney injury, it is likely that uCypA also provides similar information in other clinical situations.

Currently, the diagnosis of AKI is based on indicators of kidney function, including sCr and urine output [34]. However, kidney injury can occur in the absence of an increase in sCr, which represents an emerging condition, called subclinical AKI, an early step in the spectrum of kidney injury [35]. However, no injury biomarker is currently in clinical use, and there is an unmet medical need. NGAL is one of the best characterized AKI biomarkers. It is a 25 kDa protein expressed by many cells and is secreted in the kidney by distal tubular cells in response to ischemic or toxic injury [36], in a “severity-dependent” manner. uNGAL may increase from 3 h after the tubular insult, peaking at 6 to 12 h depending on the intensity of the insult [34,37]. Clinical studies have presented NGAL as a biomarker to predict AKI, and for early diagnosis, duration and short- and long-term outcome predictors and recovery predictors [34]. Specifically, uNGAL has been proposed as a suitable biomarker for early detection of AKI in patients undergoing cardiac surgery [19]. However, the diagnostic cut-off values for urinary or plasma NGAL are yet unclear [38].

CypA is an abundant intracellular protein expressed by numerous tissues that could be released by different cell types in response to diverse stimuli. In fact, the release of CypA has been observed in vascular smooth muscle cells (VSMCs) in response to oxidative stress and rapamycin, in macrophages treated with LPS or high glucose, in myocytes in response to hypoxia/reoxygenation and in activated platelets [39,40,41,42,43]. The cellular mechanism that induced CypA release seems to be associated to cell death, but whether it may represent specific forms of cell death is unclear. In Jurkat cells, CypA release was reported as a marker of necroptosis [14], an observation that triggered the present research. More recently, it was also linked to apoptosis in VSMCs [40]. The kidney has relatively high levels of CypA, especially in proximal tubule epithelial cells [15,16] and we now report that CypA is released by tubular cells in response to hypoxia and different lethal stimuli, but it does not seem to be specific of a unique cell death pathway. Recently, it was described that tubular cells release other proteins when exposed to cell death inducers, such as HMGB1 and IL-33 [44,45], but their clinical relevance is unknown. The cellular processes that mediate CypA release are unclear and may depend on the stimulus, as observed for VSMCs, and differ for sublethal or lethal stimuli [39,40]. Our experiments also suggest that CypA release is dependent of the loss of membrane integrity since it was not observed at early time points during apoptosis induced by TTI. In this regard, we hypothesize that intracellular CypA levels remained constant while extracellular CypA levels increased because of the presence of two-cell populations; cells that remain alive and attached to the plate, which do not release CypA and, thus, intracellular CypA levels remain stable, and dying cells that detach from the plate and release CypA. 

CypA can be freely filtered by glomeruli, so increased urinary levels could reflect increased serum levels or increased excretion from injured tubular cells [19]. In this regard, uCypA could be an early AKI marker [19,46]. We now observed that uCypA increased in clinical situations characterized by kidney IRI, such as renal artery clamping during IRI-NSS and living donor IRI-KT. We took advantage of clinical situations in which urine output and sCr may not represent kidney injury to characterize uCypA as a marker of AKI independent from kidney function. Thus, in NSS, the combination of a healthy kidney and limited ischemia usually results in stable sCr and diuresis, while in living donor transplantation, any ischemia-induced kidney dysfunction is obscured by the fact that even when injured, the kidney graft provides higher glomerular filtration than non-functioning native kidneys. Both clinical situations displayed a similar uCypA time-course, characterized by a very early increase (just after surgery was completed) and a progressive increase thereafter that is consistent with our current understanding of cell death dynamics in AKI [6]. Thus, an immediate wave of cell death in direct response to the insult may be followed by a second wave of cell death at 48–72 h fueled by local inflammation and lethal cytokines produced in response to the original insult and to the first wave of proinflammatory cell death, i.e., by RN [3,6,45]. The more limited time-course of increased uCypA in NSS with arterial clamp may represent the more limited nature of the insult, and, consequently, of kidney injury. In this regard, the magnitude of the change was larger in KT patients that in NSS with clamping (20.82 ± 6.8 vs. 10.58 ± 3.2 ng/mg at 48 h). The early post-transplant period is characterized by very high urine creatinine excretion, as not only the ongoing production of creatinine by muscle is excreted, but additionally the very high pre-transplant levels of creatinine are excreted. Thus, the normalization of uCypA by urinary creatinine may underestimate the magnitude of the change. In patients under renal replacement treatment (hemodialysis and peritoneal dialysis) elevated plasma CypA levels have been linked with systemic inflammation [43]. If, in the transplant situation, uCypA originated mainly from the circulation, this would have resulted in higher initial uCypA levels and a progressive reduction thereafter. However, in our KT population, uCypA increased progressively up to 48 to 72 h after KT, while kidneys had immediate function and sCr decreased after surgery. This is again consistent with the second wave of cell death triggered by the initial insult induction of proinflammatory RN cell death.

As increased uCypA has been observed in diabetic nephropathy. An interesting question is whether pre-existing conditions are also associated with increased uCypA [17]. This is the case for putative markers of AKI, such as NGAL and KIM-1 [33], as we confirmed in the present report for NGAL. Indeed, this might also be the case for uCypA, although we could not observe it. In any case, diabetic nephropathy is recognized to be associated with an increased rate of cell death [18,47], thus supporting a potential association of uCypA with tubular cell death.

The description of urinary markers of kidney injury that respond to NSS and KT insults, even in the absence of clinical evidence of AKI, opens the door to quantification of kidney injury in these scenarios and to future studies assessing the long-term outcome impact on kidney function of different degrees of injury. Additionally, it will allow for the assessment of the impact of different nephroprotective maneuvers aimed at decreasing kidney injury during surgery or in the immediate post-surgery period.

Some limitations should be acknowledged. The sample size was relatively small, and long-term outcomes were not evaluated. In this regard, the cut-off points used in the present study represent working cut-off points that should be better defined in future studies. Additionally, the study was not randomized. Thus, clamping was usually decided based on surgical conditions and was usually performed in larger tumors, as evidenced by larger tumor size and higher DAP and NePhRO nephrometry data. However, NSS and KT data were consistent, and this information may guide the design of future observational or interventional studies.

## 5. Conclusions

CypA is released by kidney tubular cells exposed to diverse lethal stimuli inducing different forms of cell death, suggesting that it may clinically be useful in diverse conditions associated with AKI. In this regard, increased uCypA levels were found in clinical situations of IRI-induced kidney injury in which the functional impact of kidney injury could not be reliably assessed, and provided information different from uNGAL, uCypA appearing more sensitive to IRI (Figure 9). Thus, current AKI diagnosis requires a decreased kidney function, resulting in increased sCr or decreased urinary output (Figure 9A). However, sCr may increase for functional reasons, in the absence of kidney injury (Figure 9B). By contrast, in unilateral IRI resulting from clamping of renal artery in NSS, the contralateral kidney provides still enough kidney function to minimize changes in sCr despite injury to the clamped kidney (Figure 9C). Under these conditions, we observed that uCypA was a more sensitive marker of IRI-induced kidney injury than sCr or classical markers of injury, such as NGAL. In NSS without clamping there is no IRI and no IRI-related changes in uCypA (Figure 9D). In KT, due to the virtually inexistent kidney function at baseline, graft IRI is associated with decreasing sCr levels, despite graft kidney injury, as injured kidneys still have better function than native kidneys (Figure 9E). Again, uCypA provided evidence of kidney injury despite decreasing sCr and non-informative uNGAL. Thus, uCypA is a potential biomarker of kidney injury even in patients not meeting current diagnostic criteria for AKI, that are based on functional impact.

## Figures and Tables

**Table 1:** Characteristics of NSS patients without and with arterial clamping.

	NSS without Arterial Clamp (*n* = 13)	NSS with Arterial Clamp (*n* = 39)	*p* Value
Age (years, mean ± SD)	65.50 ± 8.97	60.30 ± 11.50	0.155
Female sex	5 (38.4%)	10 (25%)	0.278
Right kidney tumor	4 (30.7%)	19 (48.7%)	0.235
Tumor size * (mm, mean ± SD)	22.5 ± 8.93	31.3 ± 8.93	0.006
**Nephrometric scores**			
RENAL (median (IQR)) [25]	6 (1.00)	6 (2.00)	0.521
PADUA (median (IQR)) [26]	7 (0.00)	8 (2.00)	0.071
C.INDEX (mean ± SD) [27]	3.25 ± 1.47	2.52 ± 0.92	0.141
DAP (median (IQR)) [28]	5 (2.00)	6 (2.00)	0.021
NePhRO (median (IQR)) [29]	6 (2.00)	8 (2.00)	0.011
ABC category [30]			
1	5 (38.4%)	8 (20.5%)	0.124
2	7 (53.8%)	23 (58.9%)	
3H	1 (7.6%)	2 (5.1%)	
3S	0	6 (15,3%)	
RPS [31] <50%	3 (23%)	7 (17.9%)	1.000
Mayo score (median (IQR) [32]	3 (2.00)	3 (3.00)	0.468
Ischemia time WIT (min, mean ± SD)	0	23.83 ± 8.38	
KDIGO criteria for AKI	1 (7.69%)	4 (10.25%)	0.613

* Maximum diameter measured by CT scan. To see full characteristics of NSS patients see Supplementary Table S1.

**Table 2:** Characteristics of KT patients without and with arterial clamping.

	Kidney Donors (*n* = 6)	Kidney Recipients (*n* = 6)
**Age (years)**	58.83 ± 5.91	54.00 ± 20.43
**Female sex**	4 (66.6%)	3 (50%)
**Renal ischemia**		
CIT (min)	83.50 ± 7.60	
WIT (min)	3.17 ± 1.27	

CIT: cold ischemia time. WIT: warm ischemia time. To see full characteristics of KT patients see Supplementary Table S2.

## Data Availability

The HK2 cell line was obtained from ATTC, and The MTC cell line were obtained from Eric G. Neilson.

## Supplementary Materials

The following are available online at https://www.mdpi.com/2227-9059/9/2/217/s1, Figure S1: Uncropped gel scans, Figure S2: Uncropped gel scans, Figure S3: Conceptual figure.
