# Supplementary material for: Urinary Cyclophilin A as Marker of Tubular Cell Death and Kidney Injury
Source: Biomedicines. 2021 Feb 20;9(2):217. doi: 10.3390/biomedicines9020217 (PMC7924181; doi:10.3390/biomedicines9020217)
Supplement: Supplementary file 1 [file biomedicines-09-00217-s001.zip › biomedicines-1094170-suppl-tables (1).pdf]

Supplementary Table 1. Characteristics of NSS patients without and with arterial clamping.

	NSS without arterial clamp (n=13)	NSS with arterial clamp (n=39)	p value
Age (years, mean ± SD)	65.50 ± 8.97	60.30 ± 11.50	0.155
Female sex	5 (38.4%)	10 (25%)	0.278
Body Mass Index (kg/m^2, mean ± SD)	26.06 ± 4.09	27.80 ± 4.81	0.254
High blood pressure	6 (46.1%)	18 (46.1%)	0.989
Diabetes mellitus	3 (23%)	7 (17.9%)	0.668
Dyslipidemia	7 (53.8%)	14 (35.8%)	0.182
**Tobacco habit**			
Smoker	1 (7.6%)	13 (35.5%)	0.213
Non-smoker	7 (53.8%)	19 (48.5%)	
Ex-smoker	5 (38.4%)	9 (23%)	
CVD	2 (15.3%)	4 (10.2%)	0.584
**eGFR ($ml/min/1.73m^2$)**			
> 90	11 (84.6%)	33 (84.6%)	
60-89	0 (0.0%)	2 (5.1%)	0.859
45-59	1 (7.6%)	3 (7.6%)	
30-44	1 (7.6%)	1 (2.5%)	
Right kidney tumor	4 (30.7%)	19 (48.7%)	0.235
Tumor size* (mm, mean ± SD)	22.5 ± 8.93	31.3 ± 8.93	0.006
**Nephrometric scores**			
RENAL (median (IQR)) [40]	6 (1.00)	6 (2.00)	0.521
PADUA (median (IQR)) [41]	7 (0.00)	8 (2.00)	0.071
C.INDEX (mean ± SD) [42]	3.25 ± 1.47	2.52 ± 0.92	0.141
DAP (median (IQR)) [43]	5 (2.00)	6 (2.00)	0.021
NePhRO (median (IQR)) [44]	6 (2.00)	8 (2.00)	0.011
ABC category [45]			
1	5 (38.4%)	8 (20.5%)	0.124
2	7 (53.8%)	23 (58.9%)	
3H	1 (7.6%)	2 (5.1%)	
3S	0	6 (15,3%)	
RPS [46] <50%	3 (23%)	7 (17.9%)	1.000
Mayo score (median (IQR) [47]	3 (2.00)	3 (3.00)	0.468
Ischemia time WIT (min, mean ± SD)	0	23.83 ± 8.38	
KDIGO criteria for AKI	1 (7.69%)	4 (10.25%)	0.613

CVD: cardiovascular disease (stroke, ischemic heart disease, arrhythmia, peripheral vascular disease, heart failure).* Maximum diameter measured by CT scan.

Supplementary Table 2. Characteristics of KT patients: kidney donors and recipients.

	Kidney donors (n=6)	Kidney recipients (n=6)
**Age (years)**	58.83±5.91	54.00±20.43
**Female sex**	4 (66.6%)	3 (50%)
**Body Mass Index (kg/m²)**	24.76±3.70	24.73±4.33
**High blood pressure**	0 (0%)	6 (100%)
**Diabetes mellitus**	0 (0%)	0 (0%)
**Dyslipidemia**	2 (33.3%)	6 (100%)
**Tobacco habit**		
Non-smoker	5 (83.3%)	3 (50%)
Ex-smoker	1 (17.6%)	3 (50%)
**CVD**	0 (0%)	3 (8.1%)
**Renal ischemia**		
CIT (min)	83.50 ± 7.60	
WIT (min)	3.17 ± 1.27	

CVD: cardiovascular disease (stroke, ischemic heart disease, arrhythmia, peripheral vascular disease, heart failure). CIT: cold ischemia time. WIT: warm ischemia time.
