# Supplementary material for: Urinary Cyclophilin A as Marker of Tubular Cell Death and Kidney Injury
Source: Biomedicines. 2021 Feb 20;9(2):217. doi: 10.3390/biomedicines9020217 (PMC7924181; doi:10.3390/biomedicines9020217)
Supplement: Supplementary file 1 [file biomedicines-09-00217-s001.zip › Supplementary Figures (1).pdf]

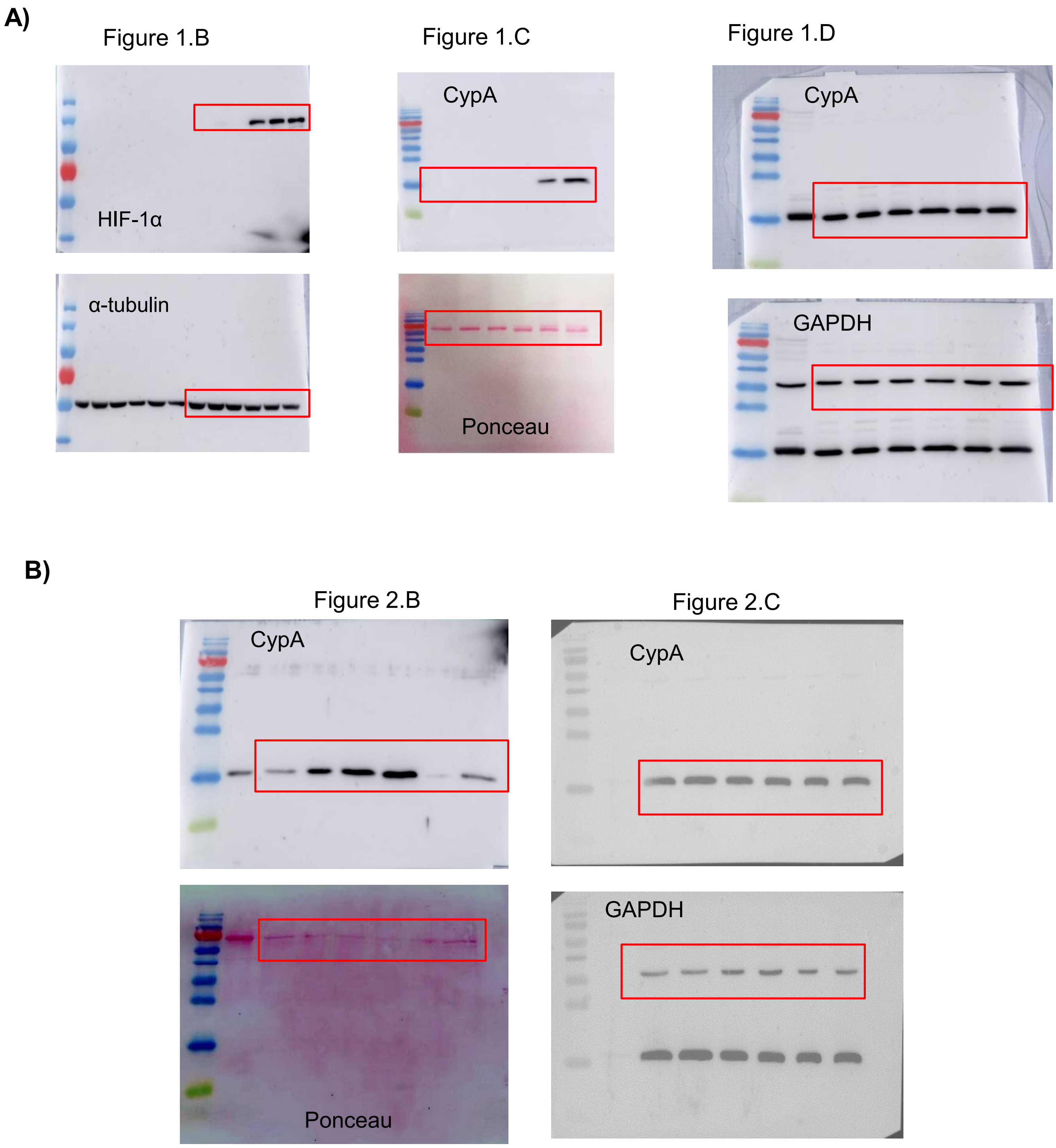

Supplementary figure 1. Uncropped gel scans merged with molecular weight marker for all presented Western blots. A) Corresponds to Fig. 1. **B)** Corresponds to Fig. 2.

A)

Figure 3.B

Figure 3.C

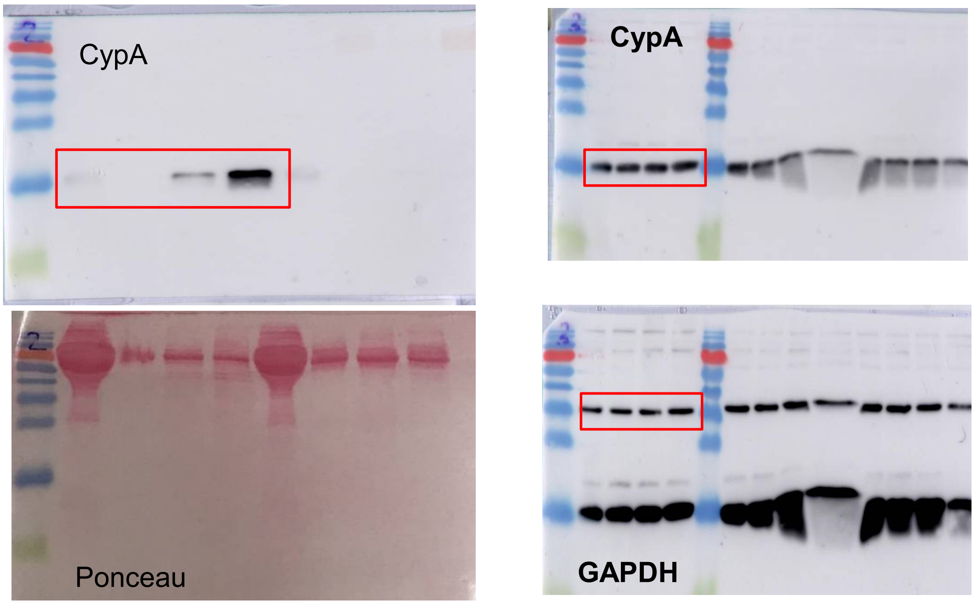

B)

Figure 4.B

Figure 4.C

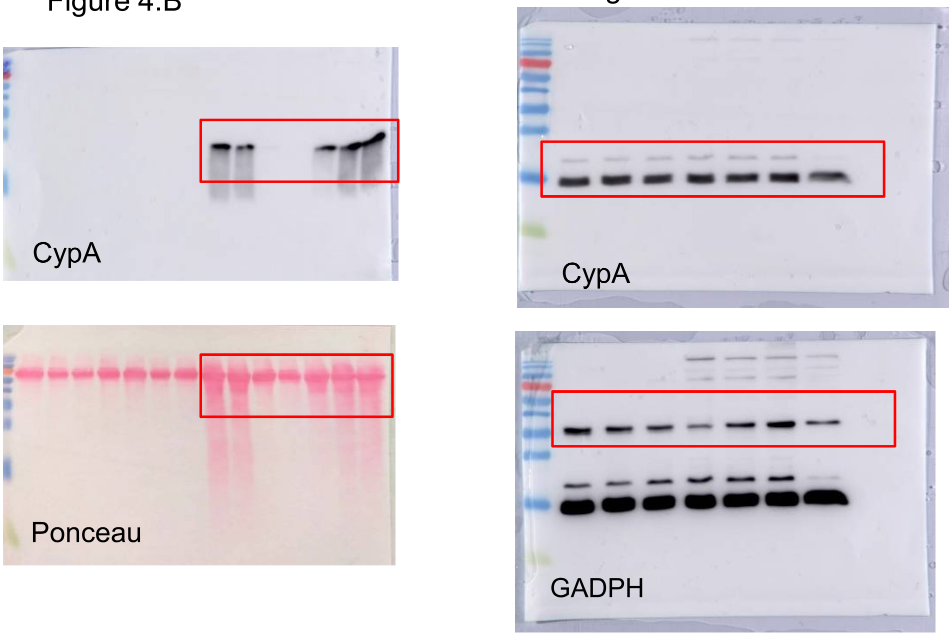

Supplementary figure 2. Uncropped gel scans merged with molecular weight marker for all presented Western blots. A) Corresponds to Fig. 3. **B)** Corresponds to Fig. 4.

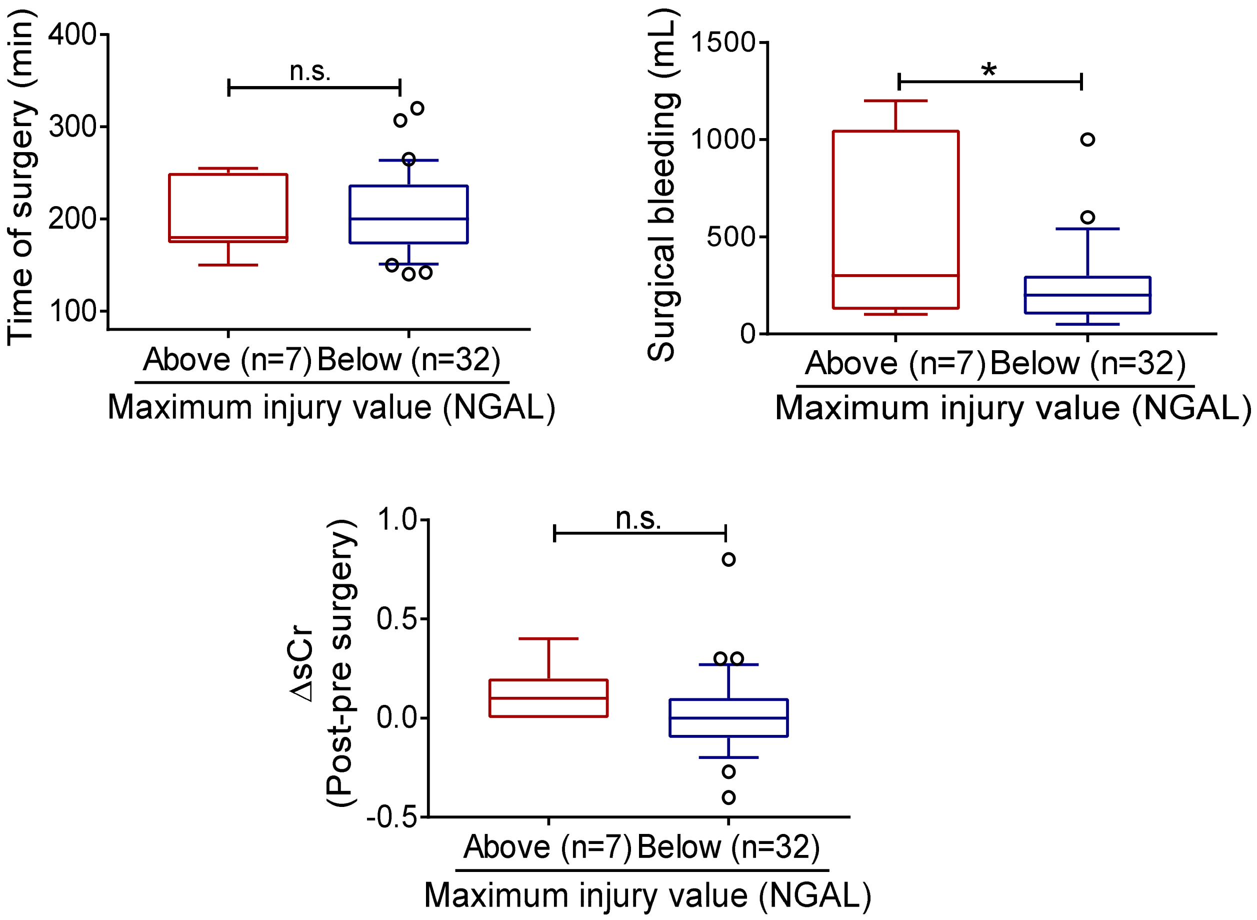

Supplementary Figure 3. uNGAL levels in NSS patients. Time of surgery, surgical bleeding and increase of sCr in patients with uNGAL above maximum injury levels. Data are presented as box plots that show the mean, 25/75 percentiles (box), and 10/90 percentiles (bars). *p<0.05.

A)

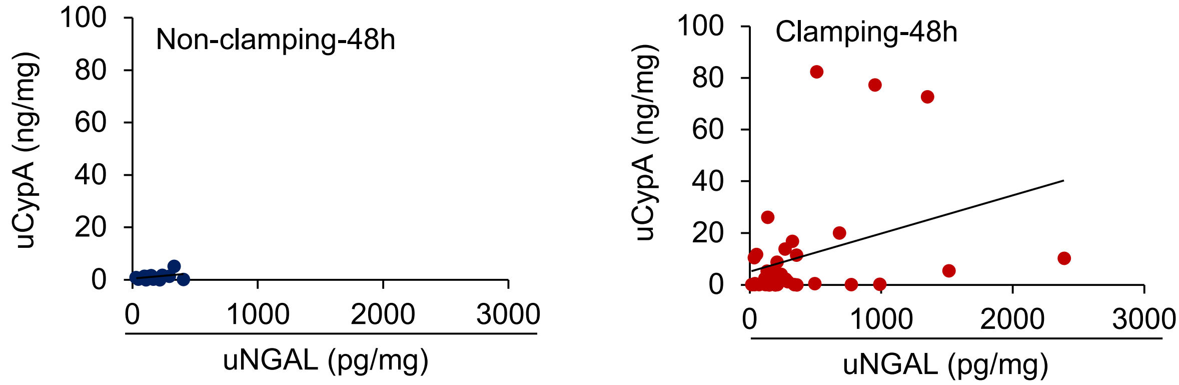

	Non-clamping 48h	Clamping 48h
R (Pearson)	0.340	0.344
p-value	0.255	0.037

B)

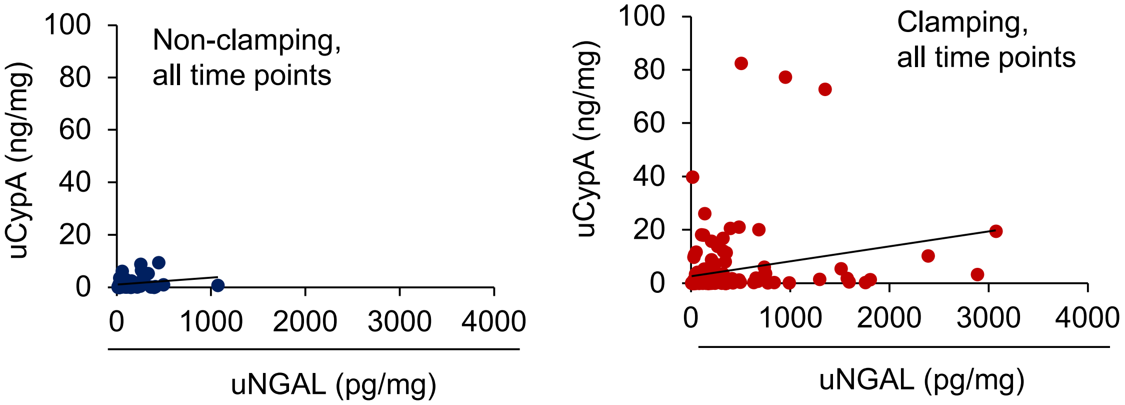

	Non-clamping, all time points	Clamping, all time points
R (Pearson)	0.167	0.238
p-value	0.241	0.002

Supplementary figure 4. Correlation between uCypA and uNGAL in NSS patients. A) At 48h post-surgery. **B)** All samples, including pre-surgery samples.
